# Optimizing larval mass-rearing techniques for *Aedes* mosquitoes: enhancing production and quality for genetic control strategies

**DOI:** 10.1051/parasite/2025024

**Published:** 2025-05-16

**Authors:** Wadaka Mamai, Cécile Brengues, Hamidou Maiga, Thomas Wallner, Anthony Herbin, Mathieu Whiteside, Simran Singh Kotla, Odet Bueno-Masso, Nanwintoum Sévérin Bimbilé Somda, Zhiyong Xi, Hanano Yamada, Chantel Janet de Beer, Jérémy Bouyer

**Affiliations:** 1 Insect Pest Control Section, Joint FAO/IAEA Centre of Nuclear Techniques in Food and Agriculture, Department of Nuclear Sciences and Applications, International Atomic Energy Agency PO Box 100 1400 Vienna Austria; 2 Institut de Recherche Agricole pour le Développement (IRAD) PO Box 2123 Yaoundé Cameroun; 3 UMR Mivegec (Maladies Infectieuses et Vecteurs : Écologie, Génétique, Évolution et Contrôle), IRD-CNRS-Université de Montpellier, Représentation IRD la Réunion – PTU 97495 Sainte Clotilde Cedex La Réunion France; 4 Institut de Recherche en Sciences de la Santé/Direction Régionale de l’Ouest (IRSS/DRO) 01 PO Box 545 Bobo-Dioulasso Burkina Faso; 5 Unité de Formation et de Recherche en Science et Technologie (UFR/ST), Université Norbert Zongo (UNZ) BP 376 Koudougou Burkina Faso; 6 Guangzhou Wolbaki Biotech Co. Ltd. 510530 Guangzhou China; 7 Department of Microbiology, Genetics, & Immunology, Michigan State University East Lansing 48824-4320 MI USA; 8 ASTRE, Cirad, INRAE, Univ. Montpellier, Plateforme Technologique CYROI 97491 Sainte-Clotilde La Réunion France

**Keywords:** Sterile insect technique, Diet ingredient, Feeding regime, Rearing-unit, Pupation

## Abstract

The quantity and quality of laboratory-reared insects are pivotal for the success of any sterile male-release program. Optimizing larval mass-rearing methods to enhance both production and quality in *Aedes* mosquitoes is essential to meet the growing demand from FAO/IAEA Member States for the sterile insect technique (SIT) as a component of area-wide integrated pest management to control or suppress disease vectors. This study was designed to identify the most effective feeding regime and schedule that maximize pupae production with a single tilt/sorting event and to evaluate an alternative larval-rearing unit. The results demonstrated that ingredient particle size, mosquito strain and feeding regime significantly influenced insect production and quality, underscoring the critical need to account for these factors in mass-rearing operations. A daily feeding regime of 0.17, 0.33, 0.67, 0.67 and 0.5 mg per larva was identified as optimal for both species (*Ae. aegypti* and *Ae. albopictus*) achieving up to 80 ± 2.5% male pupae recovery rate when sorted 48 h after the onset of pupation. Production outcomes were not compromised with the exclusion of feeding on Days 2 and 3. Furthermore, under the conditions of this study, the Wolbaki rack (Model WBK-P0003-V2) was shown to be sufficient for mass-rearing *Aedes* mosquitoes. Finally, a 4-day feeding regime was implemented in a field program on Reunion island, yielding similar pupae recovery rates and contamination as the reference regime, a significant step toward improving cost-efficiency and scaling-up the program. These findings provide valuable information for refining standard operating procedures (SOPs) for mass-rearing, thereby enhancing the efficiency and scalability of SIT programs.

## Abstract (fr)

La production en quantité et qualité des insectes en usine est essentielle au succès de tout programme de lutte basé sur le lâcher de mâles stériles. L’optimisation des méthodes d’élevage larvaire en masse pour améliorer la production et la qualité des moustiques *Aedes* est cruciale pour répondre à la demande croissante des États membres de la FAO/AIEA pour la technique de l’insecte stérile (TIS) en tant que composante de la gestion intégrée des insectes nuisibles à l’échelle des populations, visant à contrôler ou supprimer les vecteurs de maladies. Cette étude a été conçue pour identifier le régime alimentaire et le calendrier d’alimentation les plus efficaces afin de maximiser la production de nymphes au cours d’un seul événement de collecte/séparation et pour évaluer une unité d’élevage larvaire alternative. Les résultats ont montré que la taille des particules des ingrédients, la souche de moustique et le régime alimentaire influencent de manière significative la production et la qualité des insectes, soulignant l’importance de prendre en compte ces facteurs dans les opérations d’élevage en masse. Un régime alimentaire quotidien de 0,17, 0,33, 0,67, 0,67, 0,5 mg par larve a été identifié comme optimal pour les deux espèces (*Ae. aegypti* et *Ae. albopictus*), atteignant jusqu’à 80 ± 2,5 % de taux de récupération des pupes mâles triées 48 h après le début de la nymphose. De plus, un régime alimentaire excluant les jours 2 et 3 s’est révélé efficace sans compromettre les résultats de production. En outre, dans les conditions de cette étude, le rack Wolbaki (modèle WBK-P0003-V2) s’est avéré être suffisant pour l’élevage de masse des moustiques *Aedes*. Enfin, le régime alimentaire sur 4 jours a été transféré avec succès dans un programme de terrain à l’île de la Réunion, montrant un taux de récupération et de contamination des pupes mâles similaire au régime de référence, un grand pas pour amplifier la production et améliorer la rentabilité de ce programme. Ces résultats fournissent des informations précieuses pour affiner les procédures opérationnelles standard (POS) pour l’élevage à grande échelle des moustiques *Aedes*, améliorant ainsi l’efficacité et la montée en échelle des programmes TIS.

## Introduction

Mosquitoes pose a significant threat to both human and animal health by transmitting a wide range of pathogens. Within the *Aedes* genus, *Aedes aegypti* and *Ae. albopictus* are the most invasive [4, 18] and serve as major vectors for the transmission of dengue, chikungunya, yellow fever and Zika viruses [25, 40]. Methods for controlling these mosquitoes, primarily based on chemical insecticides are losing their effectiveness due to the development of resistance [34]. Moreover, these chemical methods raise public concerns due to their toxicity, lack of specificity, and the presence of residues in food and the environment [1]. Therefore, sustainable and environmentally friendly approaches to manage these vectors are needed.

Drawing on the success of the sterile insect technique (SIT), which has been used to eliminate or suppress agricultural and livestock pests such as the New World screwworm *Cochliomyia hominivorax* [23], the tsetse *Glossina austeni* [43] and the Mediterranean fruit fly *Ceratitis capitata* [14], the Food and Agricultural Organization of the United Nations (FAO) and the International Atomic Energy Agency (IAEA) are actively working to develop and expand the application of SIT for disease vector mosquito management. In recent years, there has been renewed interest from governments, municipalities and private companies worldwide to apply SIT against mosquitoes [7, 8].

Significant efforts have been made to develop equipment and protocols for mosquito SIT programs, particularly at the insect pest control laboratory (IPCL) of the joint FAO/IAEA Center of Nuclear Techniques in Food and Agriculture [42]. These efforts have been extended globally, with SIT pilot projects in countries such as Brazil, China, Italy, Mexico, Singapore, South Africa, Spain and the United States that are optimizing mosquito mass-rearing for SIT projects. For instance, the IPCL developed a specialized larval rearing unit designed to optimize mosquito production [2, 3, 29]. Furthermore, to address the high cost and limited availability of the bovine liver powder, a key ingredient in mosquito rearing, an insect-based diet alternative was developed, reducing the cost by up to 50% [5, 30]. These innovations and advancements led to the first *guidelines for mass-rearing Aedes mosquitoes* [15], with a maximum production of up to 300,000 male pupae per rearing rack unit over 5 consecutive days of tilting and sex-sorting events, representing 64% of male pupae [29]. However, the subsequent larval rearing and sex-sorting events after the first tilting pose increasing challenges, not only in terms of management, but also in maintaining the quality of the sorted pupae. Furthermore, the standard protocol involves daily feeding, including weekends, which can be costly and difficult to sustain over long periods, particularly for countries with limited budgets and workforce. It is therefore essential to identify cost-effective strategies to enhance both the quantity and quality of mass production [27].

Historically, research on mosquito rearing has primarily been focused on the nutritional value, availability and stability of diet ingredients, often overlooking factors such as diet utilization efficiency [35]. Critical factors such as the form of the diet (liquid or solid), diet particle size, appropriate daily food quantity per larva tailored to their developmental stage to ensure synchronized larval development and pupation have been underexplored. Even with the “high-quality” ingredients and optimal larval densities, determining the most effective feeding regime and schedule is crucial for achieving high pupal production in a single tilting and sorting event. Most studies in the published literature evaluating *Aedes* mosquito mass-rearing procedures for SIT programs have been conducted with a low number of larvae per rearing tray [22, 36, 39], often in Petri dishes within controlled environments such as climate chambers [37] that do not translate well to conditions of medium or large-scale mass-rearing systems. As a result, meeting the daily or weekly production requirements of millions of sterile males, necessary to achieve overflooding ratios, as demonstrated in mark-release-recapture studies [41], remains a significant challenge. Many countries currently conducting SIT pilot trials [7, 8] are facing difficulties in increasing sterile male production capacity. Therefore, there is a pressing need to understand how target species can be efficiently mass-reared in large numbers and with high quality.

In addition to optimizing mosquito production, continuous efforts to improve existing technologies are imperative to further enhance the economic viability and sustainability of SIT programs by reducing costs and increasing efficiency. In this context, Guangzhou Wolbaki Biotech Co., Ltd., a Chinese Company, has developed a new larval mass-rearing rack prototype (Model WBKP0003-V2) inspired by the FAO/IAEA model. This new rearing unit is currently used in China as part of an operational IIT/SIT program for mass-rearing *Ae. albopictus*. However, the development of new equipment does not guarantee the same efficiency across all mosquito species.

The objectives of this study were to optimize larval feeding conditions for *Aedes* mosquitoes and evaluate the performance of a new rearing unit, with the ultimate goal of establishing efficient practices to meet the demands of medium- to large-scale release programs. For *Ae. albopictus*, the study specifically investigated the influence of diet particle size, mosquito strain, feeding regime and sex-sorting schedule on pupation rates and male quality. For *Ae. aegypti,* the focus was on assessing the impact of larval feeding frequency and comparing the efficiency of the Wolbaki™ rearing unit with the FAO/IAEA aluminium unit. Finally, we tested a 4-day feeding regime in a field program on Reunion Island in comparison to three other regimes routinely used in that program. Ultimately, this study aimed to contribute to refining the guidelines for mass-rearing *Aedes* mosquitoes, while providing valuable insights into the key factors influencing the production of high-quality male mosquitoes for SIT applications. Additionally, it provides strategic procedures for testing locally available larval diets to enhance rearing efficiency and scalability.

## Material and methods

### Mosquitoes and experimental conditions

This study utilized three strains of *Aedes* mosquito species reared at the IPCL, Seibersdorf, Austria: 1) *Ae. aegypti* Brazil strain*,* from Juazeiro, Brazil, provided by Biofabrica Moscamed, an IAEA Collaborative Center since 2012; 2) *Ae. albopictus* Rimini strain, sourced from Italy, provided by Centro Agricoltura Ambiente, an IAEA Collaborative Center since 2018; 3) *Ae. albopictus* triple-infected *Wolbachia* strain (carrying wAlbA, wAlbB and wPip) as described by Zheng *et al*. [48], temporarily provided by Wolbaki Biotech Co. Ltd. Guangzhou, China. The species and strains were reared following IPCL mass-rearing procedures [15] under controlled environmental conditions. The larvae were reared in a rearing room at 28 ± 1 °C with 80 ± 10% relative humidity (RH), while the adults were maintained at 26 ± 2 °C with 60 ± 10% RH. Both rooms were set to a 14:10 h light:dark (L:D) cycle, with 1-hour simulated dusk and dawn periods. On Reunion Island, the *Ae. albopictus* strain used for the experiment originated from field egg collections in the northern region at Sainte-Marie in 2014. The adults and larvae of this strain were reared at the CYROI, Saint-Denis, Reunion Island, under laboratory conditions in a climate-controlled insectary at 27 ± 2 °C, 75 ± 2% RH, and a photoperiod of 12:12 h (light:dark).

### Experiment 1: Influence of diet particle size, mosquito strain, feeding regime and sex-sorting time on pupae recovery rate and male flight ability in *Aedes albopictus* rearing

This experiment assessed the effects of multiple (4) variables through seven distinct treatments:

(i) Diet particle size: ground *vs*. unground black soldier fly powder (BSF) (focusing on the BSF component since tuna meal and brewer’s yeast are already finely ground).
(ii) Mosquito strain: comparison of *Ae. albopictus*, Rimini strain from Italy *vs*. *Ae. albopictus* triple-infected with the bacteria *Wolbachia* from Guangzhou, China.
(iii) Feeding regime: the impact of varying amounts of larval diet per day and per larva.
(iv) Sex-sorting schedule: the optimal timing for collecting pupae after the onset of pupation, either 24 h or 48 h.

Approximately 18,000 first instar larvae were hatched from eggs on Day 0 (Thursday 2:00 pm) in jars using hatching solution and reared following the protocol described in the *guidelines for mass-rearing* Aedes *mosquitoes* v.1.0 [15] with minor modifications on diet ingredients (*e.g.*, replacing the bovine liver powder with BSF) [5, 30]. On Day 1 (Friday 9:00 am), the first instars were transferred into large mass-rearing trays and fed a liquid diet consisting of 50% tuna meal, 35% black soldier larvae powder and 15% brewer’s yeast [28, 30], according to the feeding schedule described in Table 1. The trays were tilted, and sorting was performed on Day 6 (24 h after the onset of pupation) or Day 7 (48 h after the onset of pupation). The second sorting occurred 24 h after the first. Larvae, male and female pupae were mechanically separated using a Fay-Morlan glass sorter [16, 17]. Volumetric estimation of pupae was used [15]. Partial male and female pupae recovery percentages were calculated as the ratio of pupae collected to the initial number of male or female larvae, assuming an equal sex ratio of the initial larval population. Male flight ability was assessed following methods previously described by Culbert *et al*. [10] and Maiga *et al*. [26]: after sex separation, male pupae were kept in emergence cages (30 × 30 × 30 cm, BugDorm-1H; DP1000, Taichung, Taiwan) with 10% sugar. Approximately 100 males (3–4 day-old) were aspirated and transferred into a flight test device for 2 h to calculate the escape rate. Each treatment was replicated three times, and the entire experiment was repeated twice.

**Table 1:** Experimental design for the evaluation of various factors and the daily feeding schedule for *Aedes albopictus* in Experiment 1. *The standard regime used ground ingredients*. Treatments with an (*) used the same trays.

Factors evaluated	Description	Diet preparation	IAEA larval diet: quantity in mg per larva per day (volume in mL for 18,000 larvae)							
			Day 1 (Friday)	Day 2 (Saturday)	Day 3 (Sunday)	Day 4 (Monday)	Day 5 (Tuesday)	Day 6 (Wednesday)	Total	First sorting time
	Standard regime	4% (w/v)	0.11 (50)	0.22 (100)	0.44 (200)	0.44 (200)	0.33 (150)	0.11 (50)	1.65 (750)	Day 6, 11 am (24 h)
Diet particle size	Ground ingredients	6% (w/v)	0.23 (60)	0.23 (60)	0.83 (250)	0	1.26 (380)	1.26 (380)	3.81 (1130)	Day 6, 11 am (24 h)
	Unground ingredients	6% (w/v)	0.23 (60)	0.23 (60)	0.83 (250)	0	1.26 (380)	1.26 (380)	3.81 (1130)	Day 6, 11 am (24 h)
Mosquito strain	Rimini* strain	6% (w/v)	0.17 (50)	0.33 (100)	0.67 (200)	0.67 (200)	0.5 (150)	0.17 (50)	2.51 (750)	Day 6, 11 am (24 h)
	Guangzhou strain	6% (w/v)	0.17 (50)	0.33 (100)	0.67 (200)	0.67 (200)	0.5 (150)	0.17 (50)	2.51 (750)	Day 6, 11 am (24 h)
Feeding regime	Regime 1	6% (w/v)	0.22 (100)	0.44 (200)	0.88 (200 + 200)	0	0	0.44 (200)	1.98 (900)	Day 6, 11 am (24 h)
	Regime 2	6% (w/v)	0.23 (60)	0.23 (60)	0.83 (250)	0	1.26 (380)	1.26 (380)	3.81 (1130)	Day 6, 11 am (24 h)
	Regime 3*	6% (w/v)	0.17 (50)	0.33 (100)	0.67 (200)	0.67 (200)	0.5 (150)	0.17 (50)	2.51 (750)	Day 6, 11 am (24 h)
Sex sorting time after onset pupation	24 h*	6% (w/v)	0.17 (50)	0.33 (100)	0.67 (200)	0.67 (200)	0.5 (150)	0.17 (50)	2.51 (750)	Day 6, 11 am (24 h)
	48 h	6% (w/v)	0.17 (50)	0.33 (100)	0.67 (200)	0.67 (200)	0.5 (150)	0.17 (50)	2.51 (750)	Day 7, 11 am (48 h)

### Experiment 2: Impact of larval feeding frequency (with feeding excluded on Days 2 and 3) on pupae recovery rate and male flight ability in *Aedes aegypti* rearing

In Experiment 1, feeding regime 3 was identified as the most effective among the feeding regimes tested for *Ae. albopictus.* This regime was then subsequently evaluated for *Ae. aegypti* and compared with the standard regime outlined in the guidelines [15] considered as control, as well as two alternative feeding regimes where feeding was excluded on Days 2 and 3 (weekend days in this framework). The feeding schedule for these four regimes is detailed in Table 2. As in Experiment 1, partial male and female pupae recovery percentages and male flight ability were assessed. Each treatment was replicated three times per regime, and the experiment was repeated twice.

**Table 2:** Experimental design for the evaluation of feeding regimes and schedules for *Aedes aegypti* in Experiment 2.

	Feeding regimes and larval diet quantities in mg/larva/day (mL)			
	IAEA diet 4% (w/v)-daily feeding	IAEA diet 4% (w/v) 4%-modified	IAEA diet 6% (w/v)-daily feeding	IAEA diet 6% (w/v)-modified
Egg hatching	Thursday 2 pm	Friday 9 am	Thursday 2 pm	Thursday 2 pm
L1 into trays	Friday 8:30 am	Friday 2 pm	Friday 8:30 am	Friday 8:30 am
Day 1 (Friday)	0.11 (50)	0.66 (300)	0.17 (50)	0.85 (250)
Day 2 (Saturday)	0.22 (100)	0	0.33 (100)	0
Day 3 (Sunday)	0.44 (200)	0	0.67 (200)	0
Day 4 (Monday)	0.44 (200)	0.66 (300)	0.67 (200)	0.60 (180)
Day 5 (Tuesday)	0.33 (150)	0.44 (200)	0.5 (150	0.67 (200)
Day 6 (Wednesday)	0.11 (50); first sorting	0.66 (300); first sorting	0.17 (50); first sorting	0.67 (200); first sorting
Day 7 (Thursday)	Second sorting	Second sorting	Second sorting	Second sorting

### Experiment 3: Evaluation of the efficiency of the Wolbaki rearing rack compared to the FAO/IAEA aluminium rack for *Aedes aegypti* rearing

**Figure 1:**
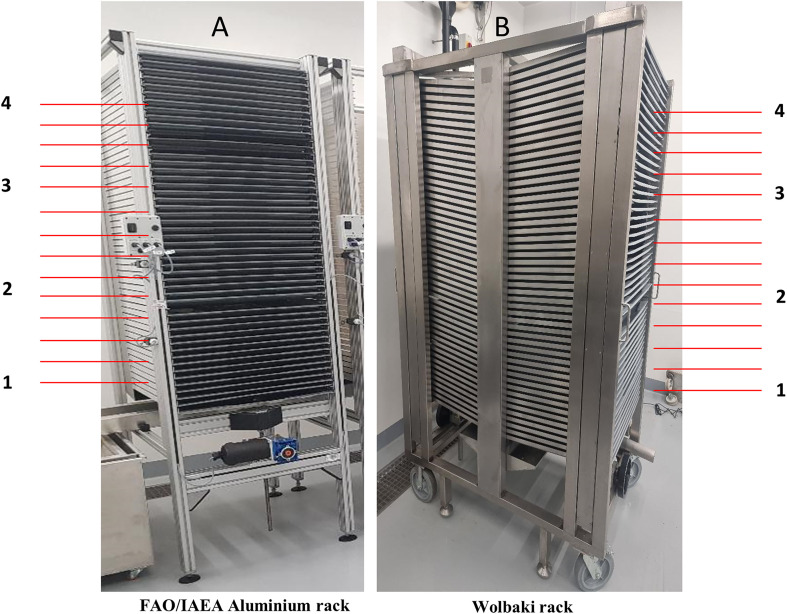
The FAO/IAEA aluminium rack (A) and the WOLBAKI rack (B) used in the experiment, with designated larval trays positions selected for sex separation. The Fay–Morlan glass sorter was used for individual tray tilting and sorting (trays 1, 2, 3 and 4), while the remaining trays were used for mixed pupae sorting with the automatic pupae sex sorter.

In this experiment, the Wolbaki rack (Model WBK-P0003-V2), which is a mechanized stainless-steel unit occupying a ground area of 1.12 m^2^ (1.35 × 0.83 × 2.1 m) and capable of holding up to 100 rearing trays (L × W × H = 70 cm × 60 cm × 3 cm; stacked in two blocks of 50 trays each) (Fig. 1B) was compared to the FAO/IAEA aluminium rack (occupying 0.71 m^2^) holding up to 50 rearing trays (L × W × H = 100 cm × 60 cm × 3 cm; Fig. 1A) for rearing *Ae. aegypti.* The comparative evaluation involved hatching eggs in jars (using cooled boiled osmosis water with 0.4% w/v larval diet), with larvae reared at 3.6 larvae/cm^2^ (or 3.6 larvae/mL) in both systems, following FAO/IAEA protocols [15]. For the FAO/IAEA rack, each tray contained 18,000 first instar larvae in 5 L of water, while the Wolbaki rack trays contained 11,000 first instars in 3 L of water. Both systems were fed a 6% IAEA liquid diet composed of 50% tuna meal, 35% BSF and 15% brewer’s yeast, according to the following feeding regimes:

- FAO/IAEA rack: 250 mL per tray on Day 1, 180 mL on Day 4, 200 mL on Days 5 and 6.
- Wolbaki rack: 150 mL on Day 1, 110 mL on Day 4, 120 mL on Days 5 and 6.

The study used 14 trays for the FAO/IAEA rack and 28 trays (14 per block) for the Wolbaki rack. Trays were tilted once on Day 7 at 9:00 a.m. and sorting was performed using either the Fay–Morlan glass sorter for mechanical sorting or the Wolbaki automatic pupae sex sorter [19, 28] (see selected trays for sorting methods in Fig. 1). Male and female pupae numbers were volumetrically estimated and partial pupae recovery percentages were calculated as described above for Experiment 1. For contamination analysis, three samples of approximately 500 and 100 pupae per sex were randomly collected for the automatic and manual sorting, respectively. These were placed into emergence cages, and after emergence and when all adults had died, were separated by sex with the naked eye (or under a stereo-microscope in cases of uncertainty). Following the contamination rates analysis, the right wings of 40 males and 40 females per sorting rack unit, sex and experiment were removed for wing length measurement using a Dino-Lite Digital Microscope (Dino-Lite AMS7025X-USB Microscope, DinoEye Eyepiece cameras, New Taipei City, Taiwan) [38]. The experiment was repeated three times.

### Experiment 4: Transfer of the 4-day feeding regime (feeding excluded on Days 2 and 3) to the field program on Reunion Island in *Aedes albopictus*

A 4-day regime (TunaBSF) using 57.5% Tuna Meal (T.C. Union Agrotech, Bang Krachao, Thailand) and 42.5% BSF powder (InnovaFeed, Paris, France) used at 4% w/v was compared to three other diet formulations and quantities used in previous phased of the field program conducted on Reunion Island (OPTIS – Opérationnalisation de la Technique de l’Insecte Stérile contre les *Aedes* vecteurs de la dengue à La Réunion) [12, 31]: Tecro1, consisting of 50% TetraMin (TETRA^©^) and 50% crushed rabbit kibble, used at 5% w/v; Tetra1 and Tetra2, consisting of two regimes of 35% TetraMin (TETRA^©^) used at 3.5% w/v (Table 3). Tetra1 was used as the reference.

**Table 3:** Diet formulations and quantities used in Experiment 4.

Food code	Ingredient 1		Ingredient 2		Dose (g/L)	Daily diet quantity (mL)					
	Name	Quantity (%)	Name	Quantity (%)		Day 1	Day 2	Day 3	Day 4	Day 5	Day 6
Tecro1	TetraMin	50	Rabbit kibble	50	50	30	60	100	150	100	150
Tetra1	TetraMin	100			35	30	60	100	150	100	150
Tetra2		100			35	30	60	100	200	150	200
TunaBSF	Tuna Meal	57.5	BSF	42.5	40	240	0	0	240	160	240

The FAO/IAEA aluminium rack described above was used for this experiment. For hatching and larvae development, filter papers containing 5-week-old eggs were gently brushed off. Three sub-samples of 500–1,000 eggs were used to confirm the hatch rate of the particular egg batch. Based on the egg hatch rate, egg batches corresponding to 14,600 first instar larvae per tray were estimated, following the method described by Zheng *et al.* [47], weighed and then hatched separately in 150 mL plastic jars filled with 100 mL of liquid diet (0.4% w/v larval diet). After hatching, the contents of jars (first-instar larvae) were transferred into mass-rearing trays (L × W × H = 100 cm × 60 cm × 3 cm, Glimberger Kunststoffe GmbH, Vösendorf, Austria) containing 5 L of water (added 1 day before the addition of larvae to allow the water temperature to adjust to room temperature and dechlorinate). Larvae were fed according to Table 3.

For sex separation of pupae, male pupae were collected on the 6th day after hatching and females the next day, and then sexed using a mechanical pupae sex sorter (Guangzhou Shanda Technology Service Co. Ltd., Guangzhou, China), following the routine procedures used in this program, where only the males collected on Day 6 are used for release, and the females collected on Day 7 for the colony. The male and female contamination rates were estimated on a sample of 500 individuals under a stereomicroscope. Male and female pupae numbers were estimated volumetrically using a modified tube following FAO/IAEA protocols [15].

### Statistical analysis

Data were analyzed using R Software, version 4.3.2 (R Development Core Team 2008) along with RStudio, version 2024.10.31 environment (RStudio, Inc. Boston, MA, USA, 2016). Binomial generalized linear mixed models (GLMMs) fit by maximum likelihood (Laplace Approximation) were used, with partial pupae recovery rate, contamination rate, and male flight ability as response variables, diet size particle, mosquito strain, sorting time, feeding regime and rack prototype as fixed effects and the replicate and experiment as random effects. A Gaussian linear mixed-effects model was used with male and female body size assigned as response variables, rack prototype as a fixed effect and replicate and experiment as random effects.

## Results

### Experiment 1: Influence of diet particle size, mosquito strain, feeding regime and sex sorting time on pupae recovery rate and male flight ability in *Aedes albopictus* rearing

**Figure 2:**
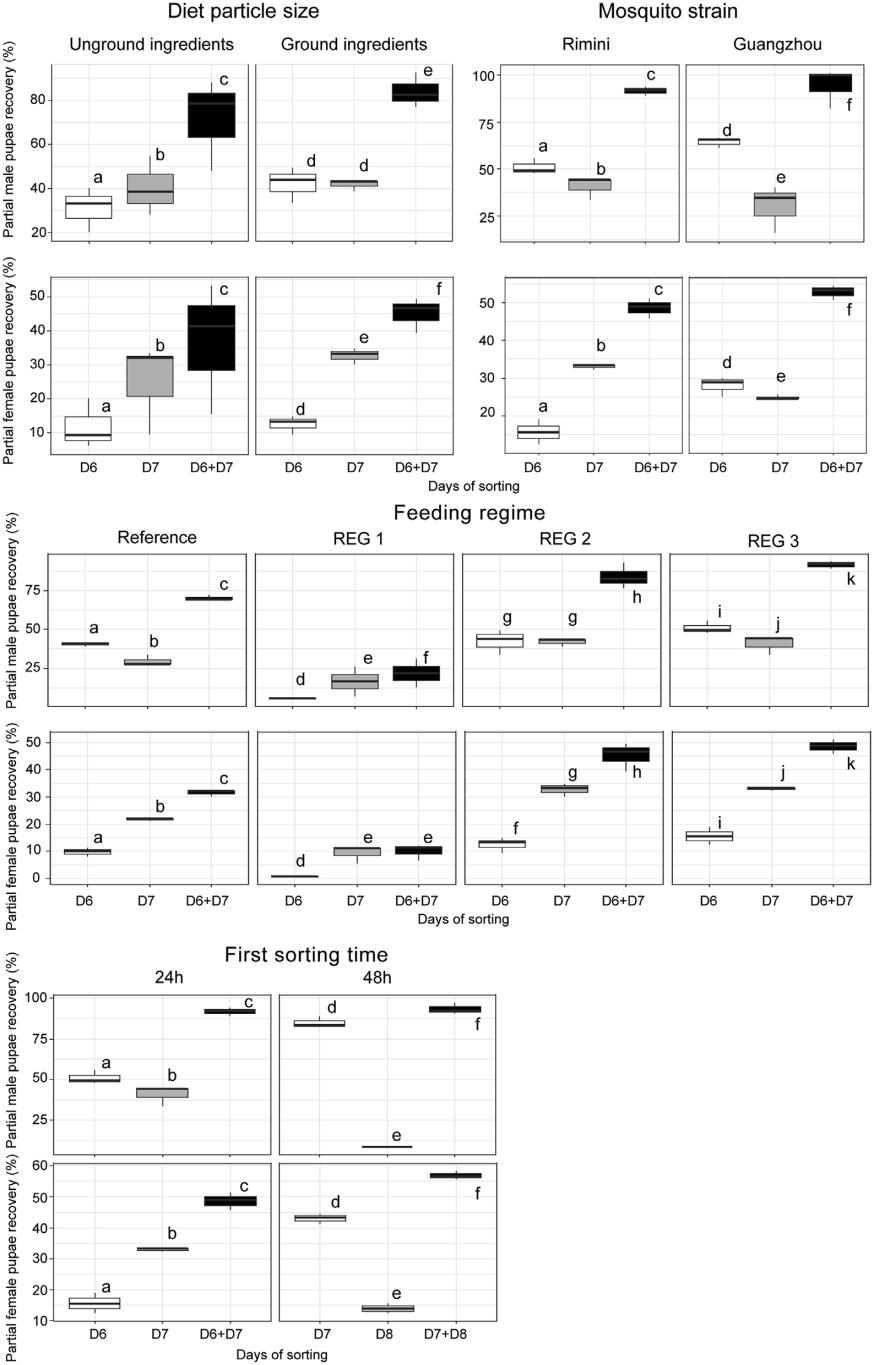
Pupae recovery rates (male and female) in Aedes albopictus rearing as influenced by diet particle size, mosquito strain, sex sorting time and feeding regime. The boxplots represent the median (line across the middle), quartiles (25th and 75th percentiles), and the minimum and maximum values (endpoints of the vertical lines). Different letters between rearing racks and sorting methods indicate statistically significant differences (*p* < 0.05).

**Figure 3:**
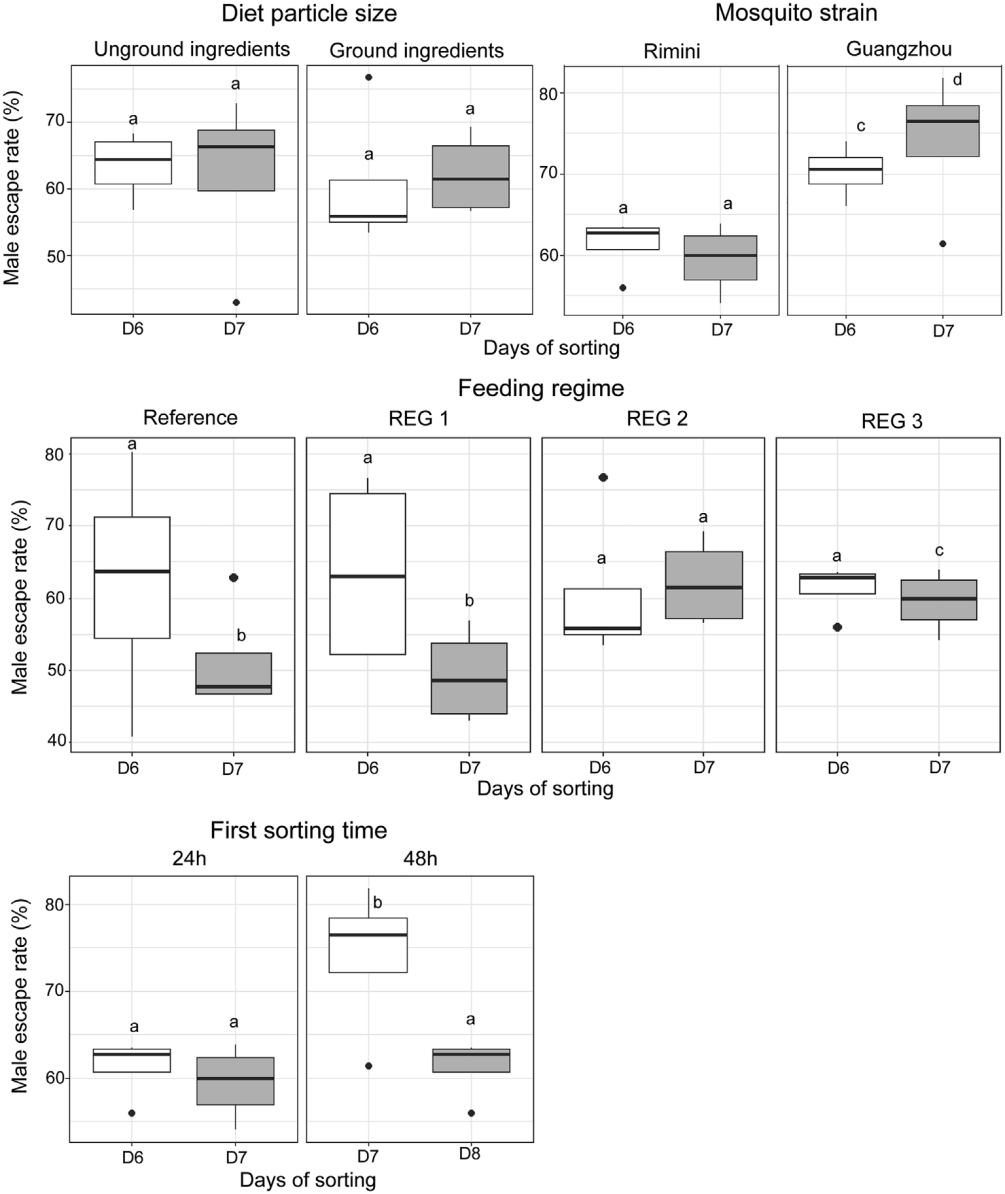
Male escape rates in *Aedes albopictus* rearing as influenced by diet particle size, mosquito strain, sex sorting time, and feeding regime. The boxplots represent the median (line across the middle), quartiles (25th and 75th percentiles), and the minimum and maximum values (endpoints of the vertical lines). Different letters between rearing racks and sorting methods indicate statistically significant differences (*p* < 0.05).

Figures 2 and 3, respectively show a summary of the partial pupae recovery rates, which correspond to the ratio of male and female pupae successfully collected as a fraction of the initial number of male or female larvae, and male escape rates under various experimental conditions, including diet particle size, mosquito strain, first sorting time, and feeding regimes. These parameters were assessed over two consecutive sorting days, Day 6 (D6) and Day 7 (D7) after egg hatching as well as the combined total for both days (D6 + D7). Mosquitoes fed with ground ingredients exhibited higher pupae production compared to those fed with unground ingredients (Fig. 2, *z* = 30.792, *p* = 2e−16 for males and *z* = 23.445, *p* = 2e−16 for females). However, no significant difference in male escape rates was observed between mosquitoes fed on ground and unground ingredients (Fig. 3, *p* > 0.05).

When comparing mosquito strains, the *Ae. albopictus-*Wolbachia infected, Guangzhou strain exhibited significantly higher pupae recovery percentage than *Ae. albopictus,* Rimini strain (*z* = 11.050, *p* = 2e−16 for males and *z* = 9.897, *p* = 2e−16 for females). Similarly, the Guangzhou strain exhibited a significantly higher male escape rate than in the Rimini strain (Fig. 3, *z* = 4.957, *p* = 7.15e−07).

Sorting conducted 48 h after the onset of pupation resulted in significantly higher pupae production compared to sorting at 24 h after the onset of pupation (*z* = 7.369, *p* = 1.71e−13 for males and *z* = 19.38, *p* = 2e−16 for females). Additionally, male escape rates were significantly higher for pupae sorted at 48 h compared to those sorted at 24 h (*p* < 0.05).

Among the three feeding regimes tested, regime 3 achieved the highest pupae recovery percentages, outperforming the standard regime as well as regime 1 and regime 2 (*p* < 0.05). While the standard regime yielded moderate pupae recovery rates, regime 1 consistently exhibited the lowest outcomes. No significant difference was observed in male escape rate among the different regimes for pupae sorted at Day 6 (24 h after first pupation). However, mosquitoes fed under regime 2 and 3 and sorted a second time on Day 7 exhibited higher male escape rates compared to those fed under regime 1 or the standard regime (Fig. 3). Overall, males sorted on Day 7 (second sorting) had lower male escape rates than those sorted on Day 6 (the first sorting).

### Experiment 2: Impact of larval feeding frequency (with feeding excluded on Days 2 and 3) pupae recovery rate and male flight ability in *Aedes aegypti* rearing

**Figure 4:**
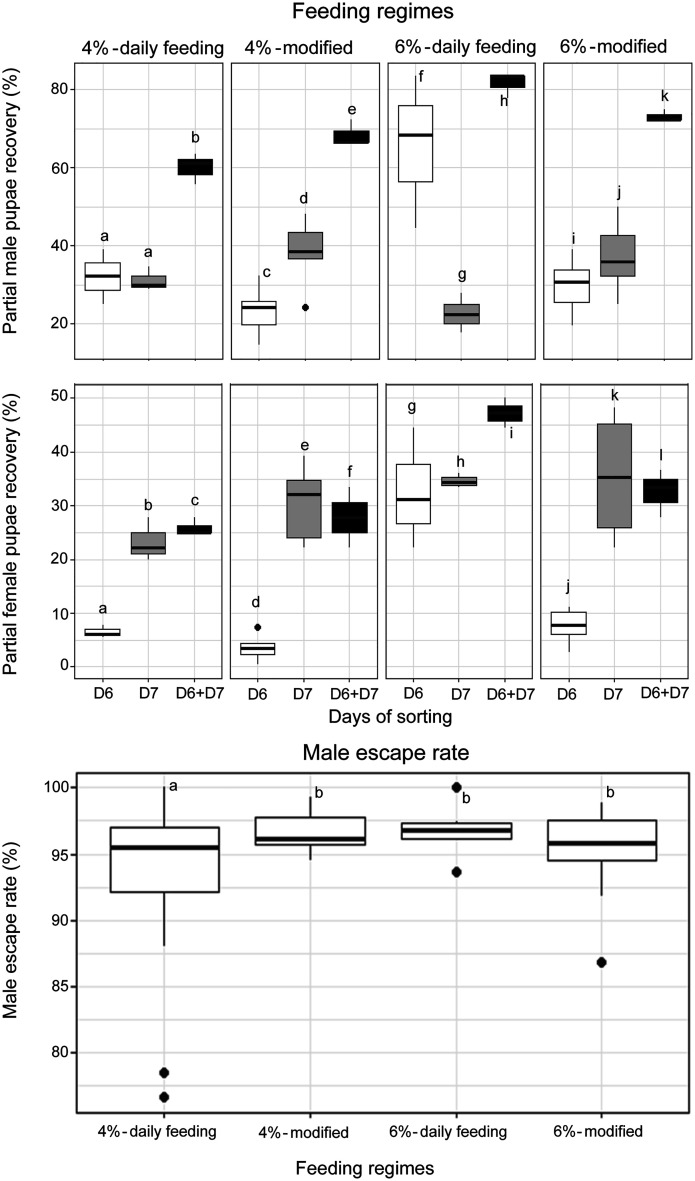
Pupae recovery rates (male and female) and male escape rates in *Aedes aegypti* rearing under different diet regime treatments. The boxplots represent the median (line across the middle), quartiles (25th and 75th percentiles), and the minimum and maximum values (endpoints of the vertical lines). Different letters between rearing racks and sorting methods indicate statistically significant differences (*p* < 0.05).

Figure 4 illustrates the effect of different diet regimes on pupation and male escape rates, focusing specifically on feeding regimes that exclude feeding on Days 2 and 3. When compared to the reference regime (4%-daily feeding), the three alternative feeding regimes (*i.e.*, 6%-daily feeding, 4%-modified and 6%-modified (both excluding Day 2 and Day 3)) resulted in significantly higher total pupae recovery percentages for both males and females (Fig. 4, *p* < 0.05). Among these, the 6%-daily feeding regime yielded the highest pupae recovery percentages both on the first collection day and cumulatively (D6 + D7). Notably, the two modified regimes (4%-modified and 6%-modified) also achieved significantly higher pupae recovery rates than the 4% daily feeding reference regime (*p* < 0.05).

Regarding male escape rates, mosquitoes fed under the 6%-daily feeding, 4%-modified, and 6%-modified regimes (excluding Days 2 and 3) exhibited similar escape rates, all of which were significantly higher than those observed under the 4%-daily feeding reference regime (Fig. 4, *p* < 0.05).

### Experiment 3: Evaluation of the efficiency of the Wolbaki rearing rack compared to the FAO/IAEA aluminium rack for *Aedes aegypti* rearing

**Figure 5:**
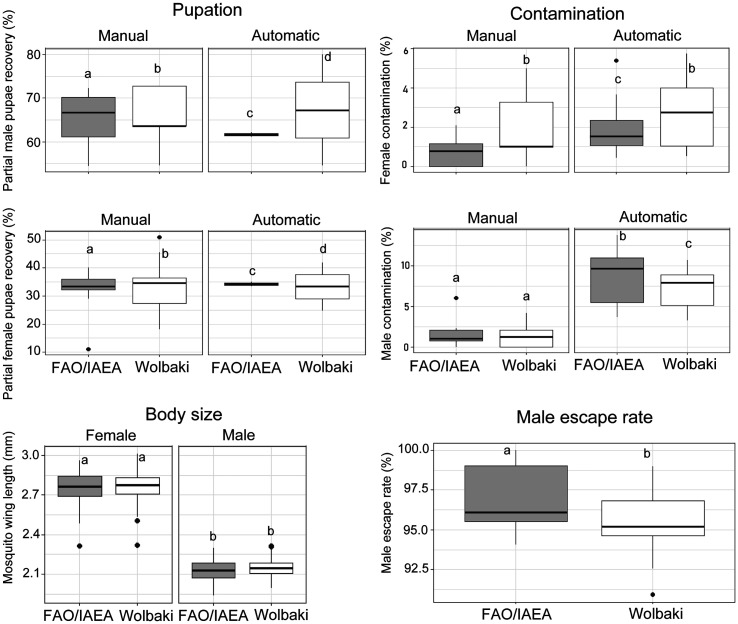
Pupae recovery rates, contamination rates, body size, and male escape rates in *Aedes aegypti* reared using two different rearing rack systems. The boxplots represent the median (line across the middle), quartiles (25th and 75th percentiles), and the minimum and maximum values (endpoints of the vertical lines). Different letters between rearing racks and sorting methods indicate statistically significant differences (*p* < 0.05).

The results for male and female pupae recovery rates, contamination rates, body sizes and male escape rates following comparison of the Wolbaki rack to the FAO/IEA aluminium rack are presented in Figure 5. The Wolbaki rearing rack consistently produced higher partial male pupae recovery percentage than the FAO/IAEA rack, regardless of whether manual and automatic sorting methods were used (*z* = 19.519, *p* < 2e−16). However, the Wolbaki rack resulted in a lower partial female pupae recovery percentage (*z* = −7.241, *p* = 4.45e−13).

In terms of contamination rates, the Wolbaki rack exhibited significantly higher female contamination rates than the FAO/IAEA rack, particularly under automatic sorting conditions (*z* = 3.882, *p* = 0.000104). Conversely, male contamination rates were lower in the Wolbaki rack than in the FAO/IAEA rack (*z* = −2.87, *p* = 0.004).

Male escape rates were significantly higher in the FAO/IAEA rack than in the Wolbaki rack (*z* = −2.366, *p* = 0.018). However, no significant differences were observed in the wing lengths of either male or female mosquitoes between the two rearing rack systems.

### Experiment 4: Transfer of the 4-day feeding regime (feeding excluded on Days 2 and 3) to the field program on Reunion Island in *Aedes albopictus*

**Figure 6:**
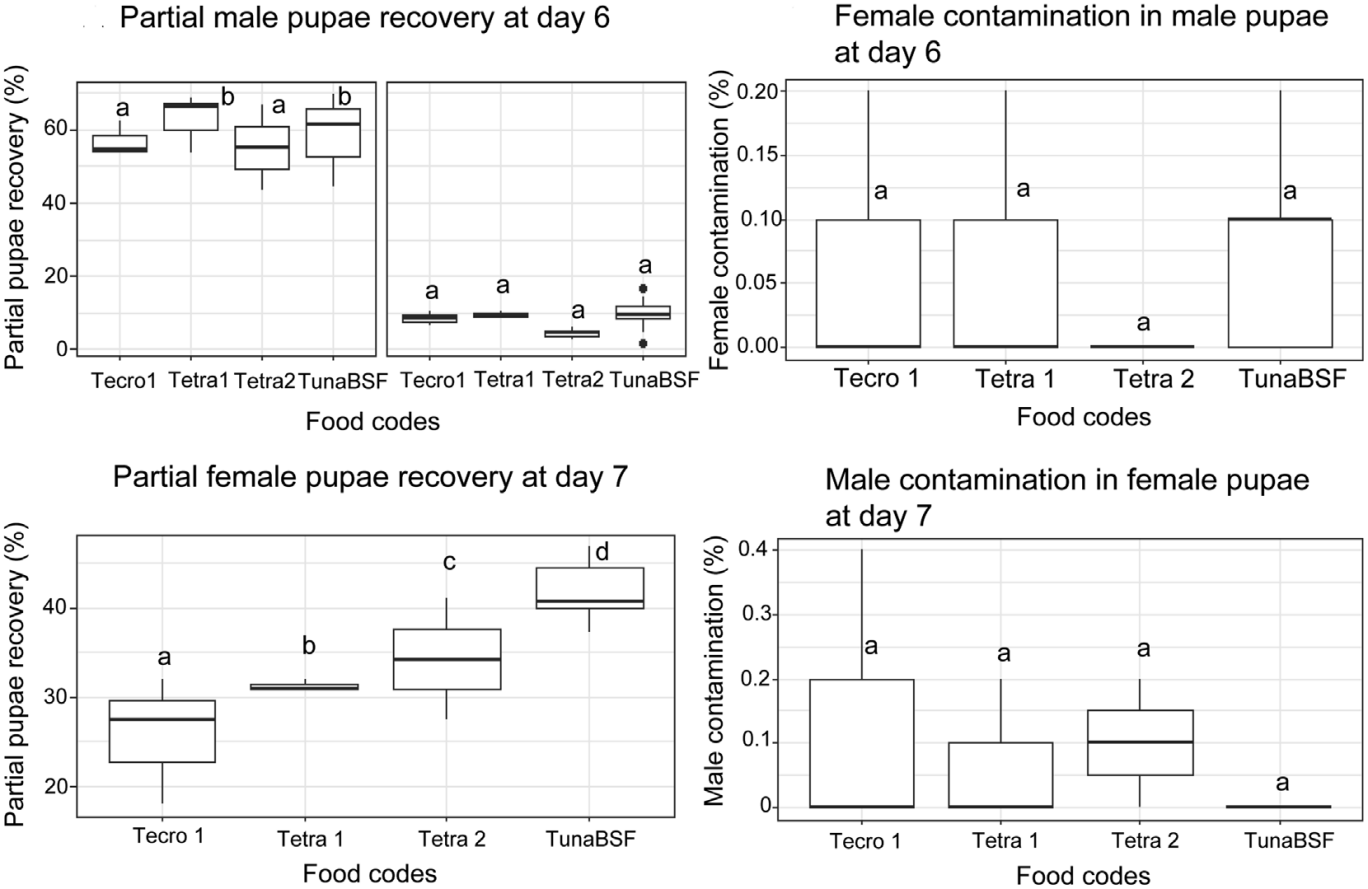
Production and contamination rates in the field program on Reunion Island in *Aedes albopictus*. The boxplots represent the median (line across the middle), quartiles (25th and 75th percentiles), and the minimum and maximum values (endpoints of the vertical lines). Values were compared to Tetra 1 as a reference point.

The feeding regime with TunaBSF that excluded feeding on Days 2 and 3 produced similar partial male pupae recovery at Day 6 to the reference regime Tetra1 (*z* = −0.271, *p* = 0.787, Fig. 6). However, it yielded the highest partial female pupae recovery at Day 7 (*z* = 3.818, *p* = 0.000135). Tecro1 and Tetra2 both lead to lower recovery rates of male pupae at Day 6 (*z* = −9.535, *p* < 2e−16 and *z* = −4.038, *p* = 5.39e−05 respectively) and lower recovery rates of female pupae at Day 7 (*z* = −11.430, *p* < 2e−16 and *z* = −2.083, *p* = 0.037247, respectively) than the reference regime.

Regarding contamination rates, no differences were observed between regimes (*p* > 0.831 for male pupae contamination at Day 6 and *p* > 0.57 for female pupae contamination at Day 7.

## Discussion

Genetic control strategies, such as the release of sterile, incompatible, or dominant-lethal males are crucial tools for controlling mosquito populations and the diseases they transmit. The efficient production of large quantities of high-quality male mosquitoes in laboratory settings is central to the success of these strategies [20]. Therefore, developing optimized and scalable rearing techniques remains a priority, requiring a multifaced approach encompassing best feeding practices and innovative rearing equipment. This study aimed to identify key factors influencing the rearing process in *Aedes* mosquitoes focusing on evaluating feeding regimes, diet particle size, mosquito strain, sorting time schedule, and rearing equipment. The findings provide important insights for optimizing *Aedes* mass-rearing protocols, particularly for genetic vector control strategies such as the SIT.

The three diet ingredients used in this study, including tuna meal, BSF larvae powder and brewer’s yeast, are shown to provide essential nutrients that effectively support larval growth and development of *Aedes* mosquitoes [5]. Specifically, the composition consisting of 50% tuna meal, 35% BSF powder, and 15% brewers’ yeast has been identified as the most effective formulation for mass-rearing *Ae. aegypti* and *Ae. albopictus* [30]. This study further confirmed the effectiveness of this diet formulation. Among the tested feeding regimes, regime 3 yielded the highest pupation percentages, outperforming the standard regime as well as regimes 1 and 2. While no significant differences in male escape rates were observed for pupae sorted on Day 6, regimes 2 and 3 resulted in significantly higher escape rates for males sorted on Day 7 compared to the standard regime. In a previous study, a pupation rate of 90%, 24 h after the onset of pupation was reported when using a diet composed of 50% tuna meal, 25% bovine liver powder, 12.5% brewer’s yeast, 12.5% squid powder, and 0.4% w/v Vitamin Mix [36]. However, this study was conducted on a small-scale, with 750 larvae per tray. Scaling up larval densities up to 4,000 larvae per tray reduced pupation rates to 75% within the same time frame [22]. In a mass-rearing facility in Italy utilizing the same rack system, Malfacini *et al*. [27] achieved male pupation rates of 27.8% and 31.1% when sorting was conducted 24 h and 48 h after the onset of pupation, respectively. Our study, conducted under mass-rearing conditions with 18,000 larvae per tray represents significant progress in optimizing production and highlights the critical importance of adapting feeding strategies to larval densities for large-scale operations.

This study also demonstrated that ground diet ingredients significantly enhanced pupation rates for both males and females, without affecting male escape rates. This suggests that finely ground diet particles are likely easier for larvae to ingest, facilitating better nutrient uptake and larval growth, leading to improved pupation rates. This result aligns with studies on mosquitoes [11, 32] and other insects, such as the yellow mealworm, *Tenebrio molitor*, where smaller particle sizes significantly improved growth and efficiency compared to larger particle sizes [33]. Merritt *et al*. [32] demonstrated that later instars of *Aedes triseriatus* consumed a proportionally higher amount of coarser material (2–50 μm), whereas early instars preferred smaller particles (<2 μm). The initial BSF powder procured contained larger particle sizes. Based on these results, the supplier has adjusted their process to provide BSF powder with finer particles prior to distribution.

The triple-infected *Wolbachia* Guangzhou strain outperformed the Rimini strain in pupation percentages for both males and females as well as in male escape rates. However, it is important to acknowledge that *Wolbachia* infection itself may confound comparisons of strain performance, as it can influence development rates and other biological processes. Dutra *et al*. [13] demonstrated that *Wolbachia* infection accelerates larval development at higher densities for both males and females. Additionally, previous studies on the Guangzhou strain revealed that the triple-infected strain developed faster from first instar larvae to pupation and from first instar larvae to adult emergence compared to both double-infected and uninfected strains [24, 46]. However, we cannot rule out that genetic differences between the Rimini and Guangzhou strains may also have played a role in the observed performance differences. These results highlight the critical role of the strain genetic background and biological characteristics in enhancing adaptability to laboratory rearing conditions, leading to higher yields and improved-quality males in mass-rearing programs.

*Aedes* mosquitoes are filter feeders with specific nutritional requirements that vary across different stages of their larval development [9, 44]. Feeding regimes are a critical component of mass-rearing. While daily feeding regimes were effective, our study found that skipping feeding on Days 2 and 3 (to coincide with weekends) did not significantly affect pupation or male escape rates in *Ae. aegypti*. This approach yielded up to 308,000 ± 10,200 male pupae per rack over two consecutive days of pupae collection in *Ae. aegypti*. Previous research on food restriction has demonstrated that *Ae. aegypti* individuals subjected to restricted diets during the larval stages exhibited a modest, but significant increase in adult lifespan compared to those provided with twice the normal amount of food [21]. While daily feeding is effective, it can be costly, requiring substantial labor, and large quantities of food. Diet and labor are known to constitute the primary costs in rearing [35]. In addition, the presence of technical staff during weekends incurs additional labor costs, which are highly context-specific and can vary significantly between countries and institutions, depending on national labor regulations and compensation frameworks. For instance, on Reunion Island, weekend work for a private company would result in an increase of up to 4% in salary cost of a technician when producing approximately 200,000 sterile males per week, and up to 13% at a production scale of 1 million sterile males. Adopting a feeding regime approach that excludes feeding on Day 2 and 3 reduces time, labor, and consequently costs, making it a more efficient alternative for mass-rearing operations. This strategy also prevents fouling of the rearing water that can sometimes occur because of overaccumulation of uneaten food by early larval stages. These findings highlight the importance of balancing productivity and cost efficiency, particularly in large-scale operations, and the potential of innovative feeding strategies to optimize the outcomes. However, future studies assessing its long-term effects on adult longevity and fitness would be highly valuable.

Sorting schedules also played a pivotal role in maximizing pupae collection and male quality during a single sorting event. Daily pupae collections are inefficient for SIT purposes, as they increase labor while reducing the quality of males produced. Using the daily feeding regime (regime 3) for rearing *Ae. albopictus*, approximately 412,450 ± 33,400 male pupae per rack could be produced over two consecutives tilting/sorting events. Alternatively, a single tilting/sorting event conducted 48 h after the onset pupation yielded 381,650 ± 11,350 male pupae per rack. Sorting 48 h after the onset of pupation resulted in significantly higher pupation percentages and male escape rates compared to sorting at 24 h, corroborating results of Malfacini *et al.* [27]. Delaying sorting allows more larvae to complete their development, ensuring a larger and more synchronized pupal cohort. These results emphasize the importance of carefully timing sex-sorting schedules to maximize both yield and quality of mosquitoes, thereby improving the efficiency of mass-rearing operations and facility management.

Rearing equipment, such as rearing racks, is another key factor influencing the efficiency and scalability of mosquito mass-rearing operations. A stainless-steel rearing rack unit developed at the IPCL of the FAO/IAEA Center has been used successfully for mass-rearing *Aedes* and *Anopheles* mosquitoes [2, 3, 29, 45]. However, to reduce the cost of the FAO/IAEA stainless-steel rack, an aluminium prototype was subsequently designed, demonstrating comparable performance (data not shown). In this study, a new model of stainless-steel rack developed by Wolbaki^TM^ was compared to the FAO/IAEA aluminium rack*.* Although the Wolbaki rack is simple and convenient for handling, it may require more time for larval feeding and tray washing due to its 100 trays, which are smaller in size than the 50 trays of the FAO/IAEA unit. Under the rearing conditions of the present study, the Wolbaki rack demonstrated a higher partial male pupae recovery rate as compared to the FAO/IAEA rack. However, it also exhibited higher female contamination rates in male pupae batches after sex separation and lower male flight ability. The reasons for these observed differences remain unclear. We hypothesize that factors such as an uneven larval distribution, asynchronous development stages, or inconsistent larval feeding could have contributed to the observed differences. Further research, particularly focusing on testing lower larval densities will help to address these issues and improve the overall efficiency of the Wolbaki rack.

The implementation of the 4-day feeding regime in a field project on Reunion Island did not negatively affect male pupae yield. Instead, it resulted in either comparable or higher male pupae recovery rates on Day 6 compared to previously used regimes. This finding is particularly relevant for optimizing resource utilization. Furthermore, the observed delay in female pupation could be advantageous in sex-separation processes by providing a clearer temporal distinction between male and female pupation times, thereby improving the sex sorting efficiency. On Reunion Island, the OPTIS program is currently expanding the area of the field trial to 175 ha to demonstrate the capacity of a boosted SIT to prevent the transmission of dengue and chikungunya, after successful small-scale field trials in Reunion Island and Spain in 2021 [6]. One objective of this program is to create all the necessary information for upscaling this technology at the operational level through a transfer to a local start-up. Demonstrating that the 4-day feeding regime – that makes it possible to avoid feeding the larvae over weekends – will be a great step to increase the cost-efficiency and upscale this program.

## Conclusion

This study demonstrated the interconnectedness and importance of various factors including diet ingredient particle size, mosquito strain, feeding regime and schedule, sorting time, and rearing equipment in determining the efficiency and scalability of mosquito mass-rearing operations. While the Wolbaki rack shows promise as an additional tool for mass-rearing mosquitoes, further studies are needed to optimize feeding regimes and larval densities to address its limitations, particularly regarding contamination rates and male flight ability. The findings provide practical insights for improving rearing practices in mass rearing conditions, particularly with regard to the 4-day feeding regime that will allow substantial cost savings.

## Data Availability

All data generated or analyzed during this study are included in this published article.
